# Supersonic plasma turbulence in the laboratory

**DOI:** 10.1038/s41467-019-09498-y

**Published:** 2019-04-15

**Authors:** T. G. White, M. T. Oliver, P. Mabey, M. Kühn-Kauffeldt, A. F. A. Bott, L. N. K. Döhl, A. R. Bell, R. Bingham, R. Clarke, J. Foster, G. Giacinti, P. Graham, R. Heathcote, M. Koenig, Y. Kuramitsu, D. Q. Lamb, J. Meinecke, Th. Michel, F. Miniati, M. Notley, B. Reville, D. Ryu, S. Sarkar, Y. Sakawa, M. P. Selwood, J. Squire, R. H. H. Scott, P. Tzeferacos, N. Woolsey, A. A. Schekochihin, G. Gregori

**Affiliations:** 1 Department of Physics, University of Oxford, Parks Road, Oxford, OX1 3PU UK; 2 Department of Physics, University of Nevada, Reno, NV 89557 USA; 3 LULI—CNRS, Ecole Polytechnique, CEA: Université Paris-Saclay; UPMC Univ Paris 06: Sorbonne Universitiés, F–91128 Palaiseau cedex, France; 4 Universität der Bundeswehr München, Neubiberg, 85579 Germany; 5 York Plasma Institute, Department of Physics, University of York, Heslington York, YO10 5DD UK; 6 Central Laser Facility, STFC Rutherford Appleton Laboratory, Harwell Oxford, Didcot, OX11 0QX UK; 7 Department of Physics, SUPA, University of Strathclyde, Glasgow, G4 0NG UK; 8 AWE, Aldermaston, Reading, West Berkshire RG7 4PR UK; 9 Max-Planck-Institut für Kernphysik, Postfach 103980, 69029 Heidelberg, Germany; 10 Graduate School of Engineering, Osaka University, Suita Osaka, 564-0871 Japan; 11 Department of Astronomy and Astrophysics, University of Chicago, 5640S. Ellis Ave, Chicago, IL 60637 USA; 12 School of Mathematics and Physics, Queens University Belfast, Belfast, BT7 1NN UK; 13 Department of Physics, School of Natural Sciences, UNIST, Ulsan, 44919 Korea; 14 Institute of Laser Engineering, Osaka, 565-0871 Japan; 15 Theoretical Astrophysics, 350-17, California Institute of Technology, Pasadena, CA 91125 USA; 16 Physics Department, University of Otago, Dunedin, 9016 New Zealand

## Abstract

The properties of supersonic, compressible plasma turbulence determine the behavior of many terrestrial and astrophysical systems. In the interstellar medium and molecular clouds, compressible turbulence plays a vital role in star formation and the evolution of our galaxy. Observations of the density and velocity power spectra in the Orion B and Perseus molecular clouds show large deviations from those predicted for incompressible turbulence. Hydrodynamic simulations attribute this to the high Mach number in the interstellar medium (ISM), although the exact details of this dependence are not well understood. Here we investigate experimentally the statistical behavior of boundary-free supersonic turbulence created by the collision of two laser-driven high-velocity turbulent plasma jets. The Mach number dependence of the slopes of the density and velocity power spectra agree with astrophysical observations, and supports the notion that the turbulence transitions from being Kolmogorov-like at low Mach number to being more Burgers-like at higher Mach numbers.

## Abstract (web summary)

Supersonic turbulence is relevant to astrophysical plasmas with their study mostly limited to numerical simulations. Here the authors demonstrate supersonic turbulence in collisional high Mach number plasma jets generated in laboratory by using high power lasers.

## Introduction

Supersonic turbulence occurs in many terrestrial^1–3^ and astrophysical systems^4–6^. For example, in giant molecular clouds (MCs), where Mach numbers can be as large as 20^6^, supersonic plasma turbulence drives the star formation rate and their initial mass function^7^. Star formation is a complex problem involving chaotic supersonic motions of the interstellar medium where self-gravity, magnetic fields, chemistry, heating, cooling, and radiative transfer all play a role^8–10^. Even the purely hydrodynamic aspects of supersonic turbulence remain poorly understood. Attempts to characterize the statistical behavior of supersonic turbulence have thus far comprised theoretical predictions^11–14^, astrophysical observations^15–17^, and hydrodynamic simulations^8,18–20^. The major challenge for the latter has been achieving an inertial range with sufficient separation between the driving and the dissipation scales to allow determination of the density and velocity power-law exponents, characteristic of the structure of turbulent fluctuations, and a popular metric for comparison between observations and simulations. Relatively few experimental studies exist, with those that do concentrating on the large velocity gradients present at the compressible turbulent boundary layers with relevance to supersonic propulsion^3,21^. To the authors’ knowledge, no experimental investigation of statistical properties of boundary-free supersonic turbulence has ever been carried out.

**Fig. 1:**
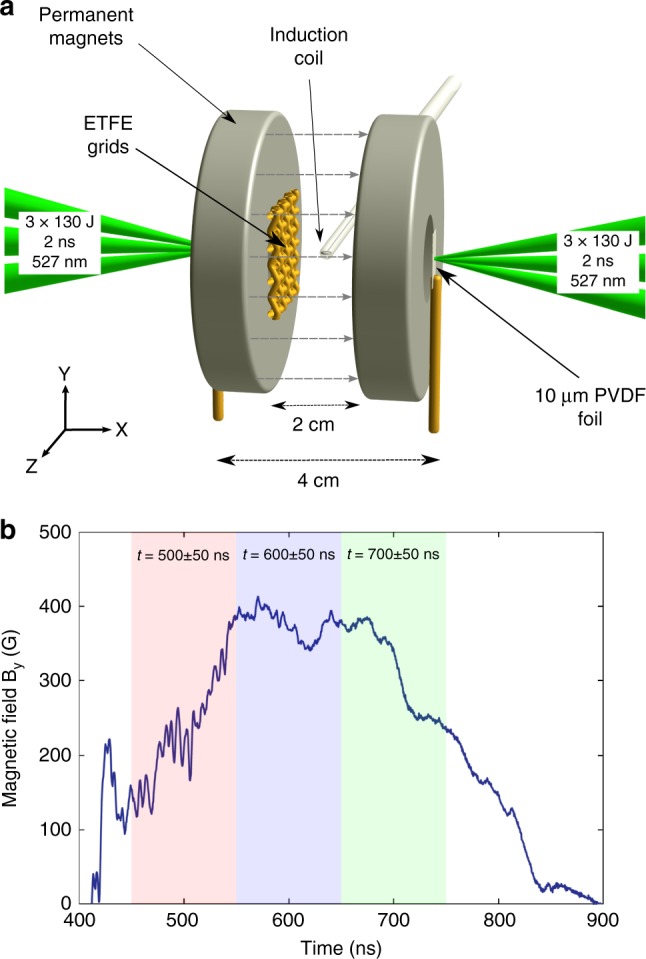
Experimental configuration and magnetic-field fluctuations. **a** Experimental configuration. Two counter-propagating supersonic jets are launched by means of optical-laser ablation of thin fluorinated plastic foils separated by 4 cm. Each foil is irradiated by three frequency-doubled (527-nm-wavelength) lasers, each carrying 130 ± 20 J of energy in a 2 ns pulse. The jets are passed through two misaligned plastic grids and collide, forming a central region of supersonic turbulence. Magnetic fluctuations, created as the magnetic field imposed by external permanent magnets (gray dashed lines) is advected by the flow, are measured with an induction coil and used to deduce velocity fluctuations. **b** Temporal evolution of the *y*-component (vertical in the top panel) of the magnetic field, as measured by the induction loop. The shaded regions represent the intervals over which the FFT was performed in calculating the magnetic-field power spectra

We provide a detailed characterization of the bulk properties of compressible turbulence in a super-Alfvénic plasma based on laboratory experiments performed with high-power lasers. We launch two counter-propagating supersonic jets by laser irradiation of thin fluorinated plastic foils (Fig. 1), with their collision forming a central region of strong compressible turbulence, primarily via Kelvin-Helmholtz shearing instabilities^22,23^. The outer-scale motions are made more chaotic by letting the jets pass through two offset grids before the collision, driving turbulence at a scale-length of roughly twice the grid spacing, or 2 mm (see Supplementary Note 1). The evolving density power spectrum is measured by means of gated Schlieren imaging, delayed with respect to the jet collision. The velocity power spectrum is probed by introducing a dynamically unimportant magnetic-field tracer, measured in the collision region by an induction loop (“B-dot probe”). Plasma density (*n*_i_), temperature (*T*_e_), ionization (Z^*^), and turbulent velocity (*V*_turb_) are obtained at different times by means of gated optical interferometry and spectroscopy, allowing calculation of the Mach number of the turbulent motions (*M*_turb_). The experiments were conducted on the Vulcan laser at the Central Laser Facility located at the Rutherford Appleton Laboratory (UK). Further experimental details are given in the Methods section.

## Results

### Measurement of the plasma conditions

**Fig. 2:**
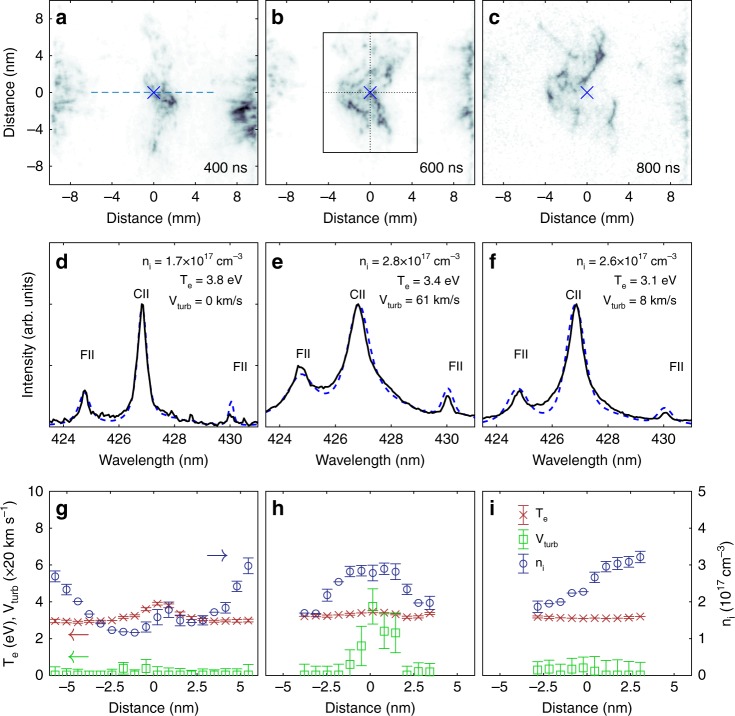
Schlieren images and optical emission spectroscopy data. Schlieren images taken at **a** 400 ns, **b** 600 ns, and **c** 800 ns after the peak of the drive laser. The box in panel **b** shows the region over which the density power spectra, shown in Fig. 3a–c, are calculated. Panels **d**–**f** show experimental emission spectra (solid black lines) from the regions marked by × in panels **a**–**c**, along with the best-fit theoretical spectrum (blue-dashed lines). The parameters given correspond to the plotted theoretical spectrum. Panels **g**–**i** show corresponding profiles of temperature (red crosses), density (blue circles), and turbulent velocity (green squares) across the region indicated by a dashed line in panel **a**, as inferred from the optical emission spectroscopy. The error bars represent a 10% deviation between the calculated and measured emission spectra

The evolution of the spatial density fluctuations measured by Schlieren imaging is shown in Fig. 2, along with the corresponding plasma properties, which have been extracted by fitting the spatially resolved emission spectrum using the collisional-radiative code PrismSPECT (details of the fitting procedure are given in the supplementary methods). The temporally resolved magnetic-field fluctuations, measured in the central region by the induction loop, are shown in Fig. 1. The ionization state of the plasma was inferred to be Z^*^ ≈ 1–2, from both optical interferometry and spectral-line fits, while the electron temperature was found to be *T*_e_ ≈ 3–4 eV. The collisional electron-ion temperature equilibration time is ≈10 ns, suggesting that the plasma quickly achieves thermodynamic equilibrium, *T*_e_ ≈ *T*_i_, with a sound speed of *c*_s_ ≈ 10–12 km s^−1^.

The Schlieren imaging and interferometry show that the jet collision occurs at ≈300 ns, implying an initial mean jet velocity of *V*_jet_ ≈ 66 ± 11 km s^−1^ and a jet Mach number *M*_jet_ ≈ *V*_jet_/*c*_s_ ≈ 6 for the plasma. We define the turbulent Mach number, *M*_turb_, characterizing plasma turbulence after the collision, as the ratio between the three-dimensional (3-D) turbulent velocity (*V*_turb_) and the sound speed, where the former is estimated from the non-thermal broadening of the carbon and fluorine emission lines^23^. We find that the turbulent Mach number increases from *M*_turb_ ≈ 0.5 ± 0.5 at *t* = 400 ns to *M*_turb_ ≈ 5.4 ± 0.8 at *t* = 700 ns. From the increase in Mach number, we conclude that the interaction of the two plasma jets continuously drives turbulence for a time much longer than the pulse duration of laser beams. The range of values of *M*_turb_ implies that the turbulence evolves from a subsonic to a highly supersonic regime as time progresses, and eventually decaying off at the very end of our measurements.

The temperature of the plasma, measured by spectral-line fitting, remains between 3 and 4 eV for all times probed and across the extent of the plasma. A radiative cooling rate of 0.5 eV/ns per ion was calculated with PrismSPECT. As shown in previous experimental work^23^, these colliding turbulent plasmas exhibit an energy balance between radiative cooling and heating, the latter presumably due to turbulent or shock dissipation. Such isothermal conditions are analogous to what is found in MCs, albeit in the astrophysical case, the balance is between cosmic ray heating and cooling via molecular-line emission^24^.

### The density power spectrum

**Fig. 3:**
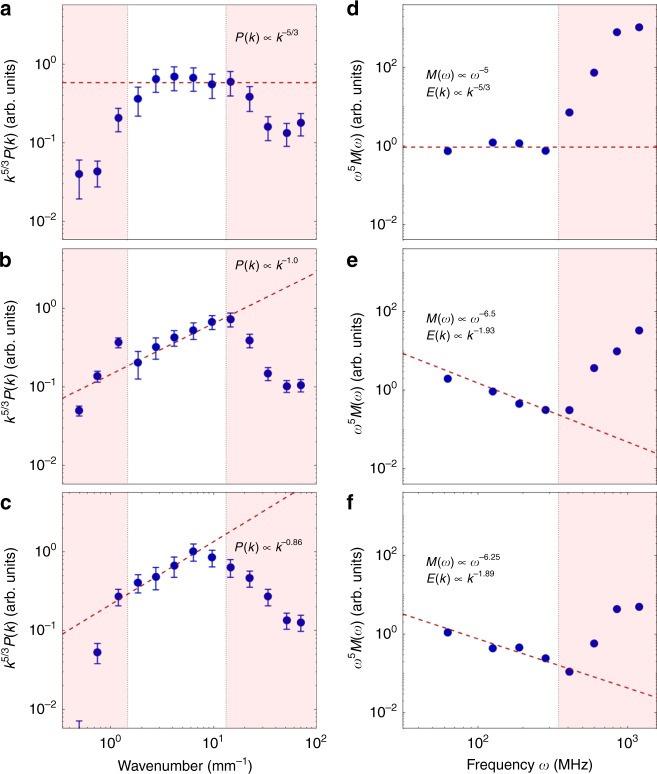
Density and velocity power spectra. The blue dots in panels (**a**–**c**) show the measured density power spectra at 500 ns, 600 ns, and 700 ns, respectively, obtained from the corresponding Schlieren images. The dashed line shows a fitted slope of the power spectrum, while the shaded regions mark the resolution limit of the imaging system (upper bound on wave numbers) and the size of the windowing function applied during the calculation (lower bound on wave numbers). The error bars show the standard deviation in the spectra based on measurements performed in four regions of the plasma—see box in Fig. 2b. The blue dots in panels (**d**–**f**) show the magnetic-field power spectra taken at 500 ns, 600 ns, and 700 ns, respectively, obtained from the induction-coil data (Fig. 1). The shaded region marks the resolution limit of the induction loop. Additional details on the calculation of resolution limits and error bars are given in the supplementary methods

We first consider density fluctuations extracted from the Schlieren intensity images, and secondly, the velocity fluctuations extracted from the time-resolved magnetic-field measurements. Assuming homogeneous ionization, the measured Schlieren signal is proportional to the integral of the density gradient along the light-path through the plasma. Thus, the two-dimensional (2-D) discrete Fourier transform of the Schlieren intensity can be related to the slope of the one-dimensional (1-D) power spectrum of the electron-density fluctuations (see supplementary methods). Figure 3a–c show the 1-D density power spectra at 500 ns, 600 ns, and 700 ns, respectively. During the initial stages of the jet collision, the turbulence is subsonic (*M*_turb_ ~ 0.5), and the spectrum is, unsurprisingly, consistent with a Kolmogorov power-law^25^, *P*(*k*) ∝ *k*^−5/3^ (where *k* is the wave number). At later times, the turbulent velocity increases and the plasma becomes increasingly supersonic, up to *M*_turb_ ~ 5.5, with the spectrum flattening to *P*(*k*) ∝ *k*^−0.86^ at 700 ns. At each time, an apparent inertial range spanning approximately a decade is achieved. The cutoff scale associated with the resolution of the measurement remains above the viscous dissipation scale, which is ≈100 nm. The similarity of the spectra at 600 and 700 ns suggests that we have fully developed, steady-state turbulence. In addition, we note that the lifetime of the plasma is much longer than the typical outer-scale turnover time, which is ≤100 ns if we estimate the outer-scale to be between 2 and 5 mm (see Supplementary Note 1). The observed flattening of the spectra with increasing *M*_turb_ is consistent with the development of fine-scale density perturbations due to shock formation, whose sharp features produce a broad power spectrum. The formation of small-scale shocks is characteristic of supersonic turbulence^7,14^.

### The velocity power spectrum

The ratio of the kinetic-energy density to the magnetic pressure is *β*_turb_ = 150, and the thermal pressure to magnetic pressure is *β*_th_ = 40. Therefore, the magnetic field is dynamically insignificant. In a poorly conducting plasma (the experimentally measured magnetic Reynolds number is *R*_m_ ~ 0.2), the power spectrum of velocity fluctuations is related to the power spectrum of the magnetic field. Thus, the magnetic field can be viewed (and diagnostically used) as a passive tracer in otherwise hydrodynamic turbulence.

The spectrum of such passive magnetic-field fluctuations, *M*(*k*), is related to that of the velocity fluctuations, *E*(*k*), by *M*(*k*) ∝ *k*^−2^*E*(*k*). This relationship is a natural consequence of the induction equation and has previously been derived for incompressible fluids^26,27^. It is also valid in a compressible fluid with an imposed external magnetic field (see supplementary methods). Extracting *E*(*k*) from magnetic-field data obtained by the B-dot probe is, however, complicated as the induction coil of the probe measures the frequency spectrum, *M*(*ω*), rather than the wave-number spectrum *M*(*k*). In the supplementary methods, we argue that, under certain physical assumptions about the structure of the turbulence and its effect on the B-dot probe, the scaling exponent of the wave-number spectrum *E*(*k*) ∝ *k*^*σ*^ can be deduced from the scaling exponent of the measured frequency spectrum *M*(*ω*) ∝ *ω*^*ξ*^ according to *σ* = −(3*ξ* + 5)/(*ξ* − 1). For reference, the Kolmogorov power spectrum *σ* = −5/3 corresponds to *ξ* = −5, and the Burgers spectrum *σ* = −2 to *ξ* = −7.

Figure 3d–f shows the power spectra of the magnetic fluctuations, at 500 ns, 600 ns, and 700 ns, respectively. During the subsonic phase of the turbulence, the power spectrum follows a *M*(*ω*) ∝ *ω*^−5^ power-law. At later times, when the turbulent velocity increases, the spectrum begins to steepen, approaching a maximum value of *M*(*ω*) ∝ *ω*^−6.5^. Utilizing our relationship between this slope and the slope of the velocity spectrum, we find initially the Kolmogorov power-law, *E*(*k*) ∝ *k*^−5/3^, which steepens, and remains close to, *E*(*k*) ∝ *k*^−1.9^ at later times, suggesting a steady state is reached. This is close to the spectrum of shock-dominated Burgers turbulence, for which theory predicts a slope of *E*(*k*) ∝ *k*^−2^, due to the development of step-like velocity profiles associated with the formation of small-scale shock structures^8,11,20^. The numerical values for the spectral exponents of the wave-number spectra of the velocity field depend on the validity of a number of (rather qualitative) assumptions required for their extraction from the frequency spectra of the magnetic field (see supplementary methods for more details). However, the steepening of the velocity spectrum is likely to be a more robust result, which we consider to be reliably established.

## Discussion

**Fig. 4:**
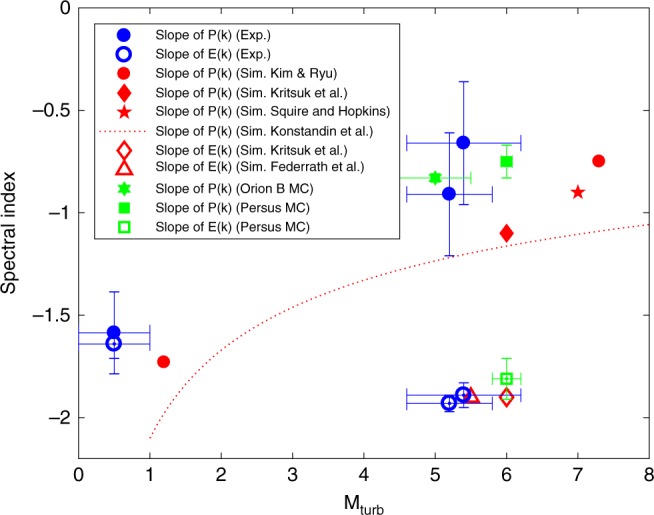
Spectral index of density and velocity turbulence. The scaling exponent of the density power spectrum, *P*(*k*) (solid markers), and the velocity power spectrum, *E*(*k*) (open markers) is plotted against the turbulent Mach number. The results from this experiment are plotted in blue, and correspond to the times *t* = 500, 600, and 700 ns. Shown in red are hydrodynamic results from Kim & Ryu^18^ (circles), Kritsuk et al.^8^ (diamonds), Squire and Hopkins^32^ (star), Konstandin et al.^33^ (dotted line), and Federrath et al.^30^ (triangle). In green are astrophysical observations of the scaling exponent of the density power spectrum of the Orion B MC^15^ (star), the density power spectrum of the Perseus MC^16,17^ (square), and the velocity power spectrum of the Perseus MC^29^ (square). The Mach number error bar is a consequence of uncertainties in the measurement of the thermodynamic conditions of the plasma (see Fig. 2). The error bars for the density spectral index show the variation across the four regions of the plasma highlighted in Fig. 2b, and the error bars in the velocity spectral index were found by shifting the Fourier transform window by 20 ns in either direction

The evolution of the spectral slope for both the density and velocity power spectra for three different times after the collision is shown in Fig. 4. At low Mach number (the subsonic case), the density fluctuations and velocity fluctuations both exhibit a Kolmogorov-like spectrum, close to *k*^−5/3^. Similar slopes for these spectra are indeed expected in nearly incompressible fluids, where perturbed density behaves like a passive scalar^28^. At higher Mach numbers, the slopes of the two power spectra diverge. The shallowing of the density power spectrum is a direct result of mass becoming concentrated in shock discontinuities^14,18^. We observe large density fluctuations (Fig. 2b, c), perhaps consistent with the formation of three-dimensional small-scale shock structures, i.e., thin sheets, and consistent also with steepened velocity spectra, discussed above—also attributed to the formation of small-scale shocks.

For context, we have also plotted in Fig. 4 the slopes of the power spectrum obtained from astrophysical observations of MCs, where large deviations from Kolmogorov turbulence are believed to exist. The Orion B MC^15^ is estimated to have a median Mach number of ~5, and observations suggest that the angularly integrated power spectrum of the column density is proportional to *k*^−2.83^, equivalent to a 1-D density power spectrum of *P*(*k*) ∝ *k*^−0.83^. Similar slopes have been found for the Perseus, Taurus, and Rosetta MCs^16,17^, which all exhibit slopes close to *P*(*k*) ∝ *k*^−0.75^. All these results fall within the error bars of our experimentally measured spectral exponents. Similarly, the velocity power spectrum in the Perseus MC^29^ is found to be *E*(*k*) ∝ *k*^−1.81^ for M ~ 6, again, in agreement with the results obtained here. The MCs discussed here are in similar hydrodynamic conditions to the experiment, with similar Mach and Reynolds numbers (Re ~ 10^5^), although the MC plasma is magnetized and most likely has a plasma *β* and magnetic Reynolds number much lower and much higher, respectively, than achieved experimentally. The effect of the magnetic field on the dynamics of MCs is a topic of active research^30^. Although magnetohydrodynamic simulations predict changes in the density power spectrum with increasing magnetic field^31^, this is beyond the scope of this work.

In Fig. 4, we also compare our results to hydrodynamic simulations of supersonic turbulence. For the velocity power spectrum, our results appear to agree with the numerical ones obtained for 3-D hydrodynamic, compressible turbulence by Kritsuk et al.^8^ and Federrath et al.^30^. For the density power spectrum, our results appear to agree with those of Kim & Ryu^18^ and Squire & Hopkins^32^. However, the experimental slopes are shallower than those predicted by Konstandin et al.^33^ and by Kritsuk et al.^8^, suggesting that noticeable differences still exist between astrophysical situations, hydrodynamic simulations, and laboratory experiments.

Thus, we have demonstrated that supersonic compressible turbulence, with a duration many times the outer-scale turnover time, can be investigated experimentally by arranging a collision of two laser-driven high-velocity plasma jets. Statistical measures of the turbulence such as the density and velocity power spectra are extracted, along with the thermodynamic properties of the plasma. Such experiments are able to provide information in addition to astrophysical observations as well as rigorous tests of numerical simulations. This opens up an avenue for the study of supersonic turbulent plasmas. Future experimental work is planned to explore the role of dynamically important magnetic fields in supersonic turbulence.

## Methods

### Experimental design

Two 10 μm PVDF (Polyvinylidene fluoride) foils separated by 4 cm are each illuminated by three 130 J, 2 ns pulse-length, frequency-doubled (527 nm wavelength) laser beams with a 200 μm spot diameter, producing a collimated jet at the rear surface of each foil. All relevant plasma parameters are given in Supplementary Table 1. The jets pass through two ring-shaped (1″ od × 5/16″ id × 1/4″) nickel-coated, N52 grade neodymium (NdFeB) magnets (K&J Magnetics, Inc., Jamison, PA), with approximately a 6000 G field in the center. Further details on the magnetic-field configuration are presented below. The spatial variation of the field is shown in Supplementary Figure 1.

The magnetic diffusion time is *τ*_D_ ~ *L*^2^/*η* ≈ 100 ns, where *L* = 2 mm is approximately the size of the plasma as it passes through the center of the disc magnet and *η* = 4.1 × 10^5^ cm^2^/s is the magnetic diffusivity of the plasma. Therefore, the magnetic field is expected to penetrate fully into the plasma during the initial stages of the experiment. After passing through the magnet, the flows are perturbed by an ETFE (Ethylene tetrafluoroethylene) grid, with a 1000 μm nominal aperture and 500 μm filaments, mounted on the surface of the magnets. The grids are misaligned so that the centers of the apertures in one grid face the vertices of the other. The misalignment of the two ETFE grids, as well as instabilities present during the collision, make the outer-scale motions as chaotic as possible, giving rise to vigorous turbulence.

Without the external magnet present, we measure fields of less than 50 G, with a similar temporal profile. This suggests that small fields are generated in the plasma by the laser-target interaction and, perhaps, by Biermann battery^21^. However, we expect that the analysis performed does not depend on the exact source of the magnetic field.

The Schlieren imaging was performed with a vertically aligned knife edge, i.e., aligned with the direction perpendicular to the bulk-flow motion. Both the interferometry and Schlieren imaging used the same optical line and were back-lit with a Photonic Solutions Powerlite Nd:YAG Laser. The laser has a wavelength of 532 nm with a ~5 ns pulse-length. Images were collected with an intensified Princeton Instruments PI-MAX CCD camera with a 4 ns gate width, synchronized to the peak of the probe laser pulse. The optical spectroscopy used a Princeton Instruments PI-MAX CCD with a 20 ns gate width.

## Supplementary information

Supplementary Information

## Data Availability

The data that support the findings of this study are available from the corresponding author upon reasonable request.
