# Supplementary material for: Supersonic plasma turbulence in the laboratory
Source: Nat Commun. 2019 Apr 15;10:1758. doi: 10.1038/s41467-019-09498-y (PMC6465398; doi:10.1038/s41467-019-09498-y)
Supplement: Supplementary file 1 — Supplementary Information [file 41467_2019_9498_MOESM1_ESM.pdf]

# Supplementary Information

T. G. White et al.

## Supplementary Figures

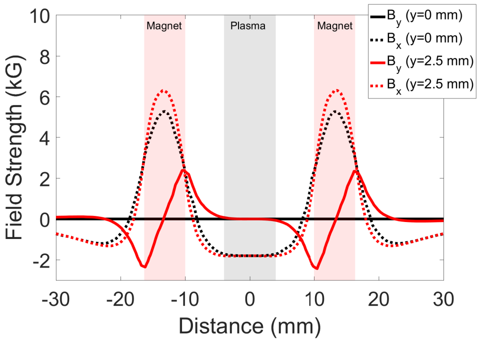


Supplementary Figure 1: **Magnetic-field strength.** Magnetic-field strength on axis and 2.5 mm off axis due to the permanent ring magnet configuration calculated using the FEMM finite element code[1]. $B_x$ is aligned parallel to the direction of flow of the jets while $B_y$ is perpendicular to the flow.

## Supplementary Notes

### Supplementary Note 1: Turbulence Scales

In table 1, we use 2 mm as the driving scale of the plasma. This value is twice the grid spacing, where the factor of two comes from magnification of the grid due to the expansion of the plasma. The distance between the plasma source and the grid is equal to the distance between the grid and the collision region. This estimate of the driving scale is further supported by the early-time density power spectrum, which deviates from a power law at a wavenumber corresponding to 2 mm, and, more phenomenologically, by the size of the initial perturbations that can be seen at early times in Fig. 2a of the main paper. The outer scale of the plasma grows with time, and, therefore, lies between the identified value, 2 mm, and the total extent of the plasma, 5 mm. This conclusion is supported by Fig. 3 in the main manuscript, where a power law holds up to increasingly larger scales at later times. We note that the viscous-dissipation scale is of the order of 100 nm, far smaller than the resolution limit of the experiment.

## Supplementary Tables

| Quantity | Origin | Value | |
|---|---|---|---|
| Average atomic weight (A) | $C_2H_2F_2$ | 10.66 a.m.u. | |
| Ion density ($n_i$) | Spectroscopy | 2 - 3$\times 10^{17}$ cm$^{-3}$ | |
| Electron density ($n_e$) | Interferometry | 3 - 6$\times 10^{17}$ cm$^{-3}$ | |
| Average ion charge ($Z^*$) | $\frac{n_e}{n_i}$ | 1 - 2 | |
| Temperature ($k_B T_e = k_B T_i$) | Spectroscopy | 3 - 4 eV | |
| Static magnetic field ($B_0$) | Permanent Magnets | 180 mT | |
| Advected magnetic field ($\Delta B$) | Induction Loop | 50 mT | |
| Thermal $\beta$ | $2\mu_0 \frac{n_i k_B T_i + n_e k_B T_e}{B_0^2}$ | 40 | |
| Turbulent $\beta$ | $2\mu_0 \frac{\frac{1}{2} A n_i V_{\text{turb}}^2}{B_0^2}$ | 500 | |
| Alfvén Velocity | $\frac{B_0}{\sqrt{\mu_0 A n_i}}$ | 2.5 km/s | |
| Sound speed ($c_s$) | $\sqrt{\frac{Z k_B T_e + \frac{5}{3} k_B T_i}{A}}$ | 10-12 km/s | |
| Debye Length ($\lambda_D$) | $\sqrt{\frac{\epsilon_0/e^2}{\left(\frac{n_e}{k_B T_e}\right) + \left(\frac{Z^2 n_i}{k_B T_i}\right)}}$ | 10 nm | |
| Coulomb Logarithm (log$\Delta$) | $\log \frac{\lambda_D}{\left(\frac{e^2}{4\pi\epsilon_0 k_B T_e}\right)}$ | 4 | |
| Ion thermal collision time ($\tau_i$) | $(4\pi\epsilon_0)^2 \frac{3\sqrt{m_i}(k_B T_i)^{\frac{3}{2}}}{4\sqrt{\pi} \log\Delta e^4 Z^4 n_i}$ | 10 ps | |
| Electron thermal collision time ($\tau_e$) | $(4\pi\epsilon_0)^2 \frac{3\sqrt{m_e}(k_B T_e)^{\frac{3}{2}}}{4\sqrt{2\pi} \log\Delta e^4 Z^2 n_i}$ | 0.5 ps | |
| Viscosity ($\nu$) | $\frac{0.96 n_i k_B T_i \tau_i}{m_i n_i}$ | 3 cm$^2$/s | |
| Resistivity ($\eta$) | $\frac{1}{\mu_0} \frac{0.51 m_e}{n_e e^2 \tau_e}$ | $10^6$ cm$^2$/s | |
| Electron-ion equilibration time ($\tau_{ie}$) | $\frac{(4\pi\epsilon_0)^2}{1.8} \frac{(m_e k_B T_i + m_i k_B T_e)^{\frac{3}{2}}}{(m_i m_e)^{\frac{1}{2}} Z^2 e^4 n_i \log\Delta}$ | ~10 ns | |
| Ion mean free path ($\lambda_{ii}$) | $\left(\frac{k_B T_i}{A}\right)^{\frac{1}{2}} \tau_i$ | 100 nm | |
| Scale Length ($L$) | | 2 mm | 200 $\mu$m |
| Velocity ($V_{\text{turb}}$) | | $< V_{jet}$ =66 km/s | 10-50 km/s |
| Eddie turnover time | $\frac{L}{V_{\text{turb}}}$ | 30 ns | 4 ns |
| Mach number ($M_{\text{turb}}$) | $V_{\text{turb}}/c_s$ | ~6 | ~1-6 |
| Fluid Reynolds number (Re) | $V_{\text{turb}} L/\nu$ | $\sim 10^5$ | $\sim 10^4$ |
| Magnetic Reynolds number (Rm) | $V_{\text{turb}} L/\eta$ | ~1 | ~0.1 |
| Viscous dissipation scale ($l_\nu$) | $L/Re^{\frac{3}{4}}$ | 100 nm | |

Supplementary Table 1: **Plasma Parameters.** Summary of relevant plasma parameters. Values correspond to data taken 600 ns after the start of the drive laser. The S.I. unit system used for all physical quantities in the above formulas and values, except the values of temperature expressed in eV and atomic mass expressed in a.m.u..

## Supplementary Methods

### Calibration of magnetic-field induction loop

The design and calibration of the induction loop ("B-dot probe") was described by Everson *et al.*[2]. The Fourier transform of the produced voltage is related to the spectrum of the magnetic field via

$$\frac{V_{\rm meas}(\omega)}{B(\omega)} = aNg\frac{\omega}{1+(\omega\tau_{\rm s})^2}(\omega\tau_{\rm s}+i), \tag{1}$$

where $\tau_{\rm s}$ is a quantity that depends on both the self- and mutual inductances of the circuit.

To calibrate the probe, a known time-varying magnetic field is generated with a Helmholtz coil, driven by a network analyzer that sweeps a reference frequency, $V_{\rm ref}(\omega)$, between 9 kHz to 500 MHz. The magnetic field at the center of the Helmholtz coil is given by

$$B(\omega) = \left(\frac{4}{5}\right)^{3/2}\frac{\mu_0}{rR_{\rm p}}[2V_{\rm ref}(\omega)], \tag{2}$$

where $r$ and $R_{\rm p}$ are the radius and resistance of the Helmholtz coil, respectively. Introducing an electrical time delay $\tau$ into supplementary eq. 1, and replacing the magnetic field with that produced by the Helmholtz coil, we obtain the frequency-resolved response of the probe[2]:

$$\frac{V_{\rm meas}(\omega)}{V_{\rm ref}(\omega)} = 2\left(\frac{4}{5}\right)^{3/2}\frac{\mu_0}{rR_{\rm p}}aNg\frac{1}{1+(\omega\tau_{\rm s})^2}\Big\{\big[\omega\tau_{\rm s}\cos(\omega\tau)-\sin(\omega\tau)\big]+i\big[\omega\tau_{\rm s}\sin(\omega\tau)+\cos(\omega\tau)\big]\Big\}, \tag{3}$$

where $N = 2$ is the number of turns in the probe, and $g = 0.5$ the gain of the differential amplifier. The probe's cross sectional area, $a$, relaxation time, $\tau_s$, and the overall delay time, $\tau$, are varied to fit the theoretical response to the calibration data. The real and imaginary components of the probe's frequency response are given in supplementary figure 2, along with a least-mean-squares fit of supplementary eq. (3) to the data. Values of the fitting parameters are given within 95% confidence bounds. Our calibration procedure showed that the frequency resolution of the probe is approximately $\omega \approx 250$ MHz. We estimate averaging effects due to finite size will impact frequencies higher than the frequency resolution of the probe.

### Extraction of density power spectra from Schlieren images

Schlieren images of the density variation in the turbulent plasma were taken at a series of times after the collision. The images have a pixel size of 50 $\mu$m and a resolution of better than 500 $\mu$m, where the resolution was calculated from the FWHM of a knife edge placed in the focus of the imaging system. We characterized the relative fluctuations in detected intensity by performing spectral analysis of the jet-interaction region. In order to distinguish small-scale density variation from large-scale inhomogeneities of the interaction region, we constructed a relative-intensity map,

$$I(x,y) = \frac{I_{\rm raw}(x,y)}{MI_{\rm smoothed}(x,y)+(1-M)I_{\rm raw}(x,y)} - 1, \tag{4}$$

where $M$ is a mask with value 1 in the center of the plasma and 0 outside the extent of the plasma. A two-dimensional Nuttall window is used to transition smoothly between the two. $I_{\rm smoothed}$ is a coarse-grained mean field calculated using a $100 \times 100$-pixel smoothing filter, where 100 pixels correspond to the approximate extent of the plasma ($\sim$5 mm).

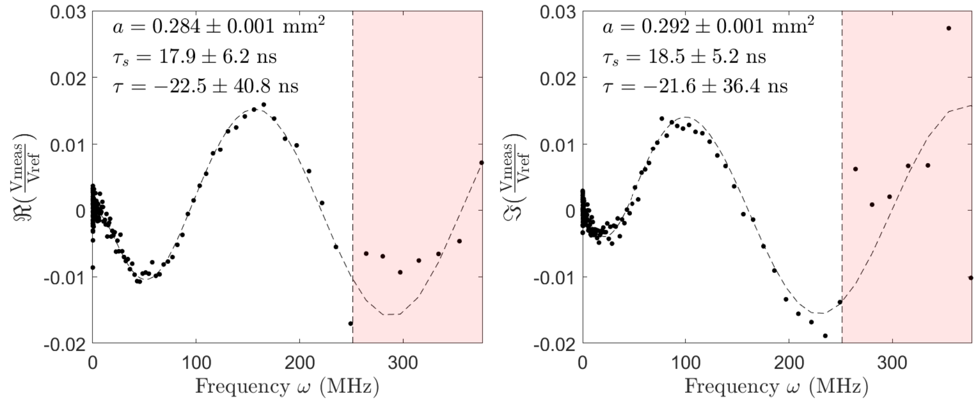


Supplementary Figure 2: **Induction-loop calibration.** The real (left) and imaginary (right) components of the frequency response of the induction loop (B-dot probe), as determined from the calibration with the Helmholtz coil. Values of the fitting parameters from supplementary eq. (3) are given with 95% confidence bounds. The deviation between the theoretical fit, dashed line, and the calibration data occurs around 250 MHz.

The power spectra were then calculated by taking average values from a logarithmically binned annular histogram of a two-dimensional fast Fourier transform (FFT) applied to the relative-intensity image. To minimize the impact of detector-based defects in the image, the median, rather than the mean, was used as a measure of central tendency for each bin: the former is more robust against the outlying values typically associated with such features. The efficacy of these methods has been successfully demonstrated previously[3] and tested by superimposing such defects on simulated Gaussian fields with prescribed spectra. Error estimates were performed by carrying out the above analysis on four regions in the image, with the standard deviation used to define the error bars in Figs. 3(a-c). The upper limit of the power spectra in Figs. 3(a-c) is defined by the resolution of the Schlieren diagnostic, $\sqrt{2} \times 2\pi/500\ \mu$m. The lower limit is instead given by the size of the windowing function discussed above, $2\pi/5$ mm.

Schlieren is an imaging technique sensitive to density variations in the plasma. The plasma is illuminated with columnated parallel light, and an imaging system is used to form an image of the plasma on the detector. An additional focusing lens and a knife edge, positioned between the plasma and the detector, block any undeflected light. Thus, the intensity of the image formed is proportional to the angular deflection acquired by the rays as they go through the plasma. For small deflections, the observed intensity of the Schlieren image $I(x, y)$ is proportional to the gradient of the refractive index along a direction orthogonal to the knife edge, $x$, integrated along the path of the light ray[4]. As the electron density, $n_e$, is much smaller than the critical density of the plasma, the intensity, $I(x, y)$, is given by,

$$I(x, y) \propto \int \frac{\partial n_e}{\partial x} \mathrm{d}z, \tag{5}$$

where $x$ and $y$ are the spatial coordinates of the image plane and $z$ is the direction normal to it.

The 2D power spectrum of the intensity is, therefore,

$$\begin{aligned}
P^I_{2D}(k_x, k_y) &= \left| \iint I(x,y) e^{-i(xk_x + yk_y)} \mathrm{d}x\mathrm{d}y \right|^2 \\
&\propto \left| \iiint \frac{\partial n_e}{\partial x} e^{-i(xk_x + yk_y)} \mathrm{d}x\mathrm{d}y\mathrm{d}z \right|^2 \\
&\propto k_x^2 \left| \iiint n_e e^{-i(xk_x + yk_y)} \mathrm{d}x\mathrm{d}y\mathrm{d}z \right|^2 \\
&\propto k_x^2 \int P^n_{3D}(k) \delta(k_z) dk_z
\end{aligned} \tag{6}$$

where $k = \sqrt{k_x^2 + k_y^2 + k_z^2}$. The right hand side of supplementary eq. (6) is simply the $k_z = 0$ cut through the 3D Fourier spectrum of the electron density, $n_e$. Thus,

$$P^I_{2D}(k_x, k_y) \propto k_x^2 P^n_{3D}(\sqrt{k_x^2 + k_y^2}). \tag{7}$$

Switching to polar coordinates ($k_x = k\cos\phi$, $k_y = k\sin\phi$) and averaging over $\phi$ gives

$$\langle P^I_{2D}(k, \phi) \rangle \propto k^2 P^n_{3D}(k). \tag{8}$$

The 3-D power spectrum of an isotropic density field is related to its 1-D density power spectrum by $P^n_{1D}(k) = 4\pi k^2 P^n_{3D}(k)$. Therefore,

$$\langle P^I_{2D}(k, \phi) \rangle \propto P^n_{1D}(k), \tag{9}$$

i.e., the angle-averaged intensity spectrum is proportional to the 1-D density power spectrum.

This relationship is confirmed by the results of numerical calculations, shown in figure 3. By the inverse discrete Fourier transform of a 3-D power law, we construct a density field on an $80\times80\times80$ grid. The desired power spectrum is scaled to give a maximum electron density of $5 \times 10^{17}/\mathrm{cm}^3$, and each Fourier harmonic is assigned a random phase. From synthetic Schlieren radiographs the power-law exponent of the angularly averaged intensity power spectrum was calculated, and compared to the exponent of the true 1-D density power spectrum. Figure 3 confirms the one-to-one relationship for the case where the intensity is proportional to the displacement of the spot at the knife edge, as assumed in equation (5). However, we note that equation (5) is an approximation, and the intensity of the image will be a more complicated function of the size and shape of the spot at the knife edge. Simulating a 100 $\mu$m FWHM Gaussian beam, with an initial position either behind or centered on the knife edge, we find a relationship within 10% of that given by equation (9). A similar result is found when simulating a circular flat-top spot. This result is due to the fact that the overlap between the spot and and knife edge is approximately linear for the small deflections achieved during the experiment.

### Magnetic field as velocity tracer

In the main manuscript, we utilize the statistical properties of the magnetic-energy spectrum to infer the properties of the velocity spectrum. The relationship between the two is a natural consequence of the induction equation,

$$\frac{\partial \mathbf{B}}{\partial t} + \mathbf{u}.\nabla\mathbf{B} = \mathbf{B}.\nabla\mathbf{u} - \mathbf{B}\nabla.\mathbf{u} + \eta\nabla^2\mathbf{B}. \tag{10}$$

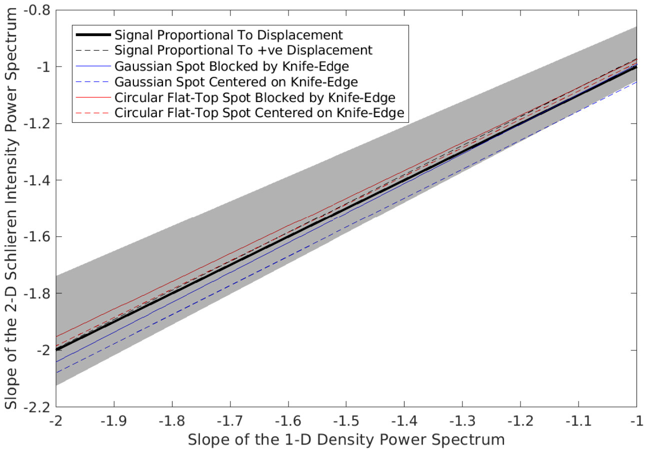


Supplementary Figure 3: **Numerical Validation of Supplementary Equation 9** For constructed density fields, as described in the text, we show the relationship between the exponents of the 1-D density power spectrum and the angularly averaged intensity power spectrum, calculated from synthetic Schlieren images. Assuming the intensity of the Schlieren is proportional the magnitude of the displacement of the focal spot, as in Eq. (5), the one-to-one relationship is recovered (black-solid line). Assuming only positive displacements contribute towards the Schlieren image, as provided by a knife-edge, changes the relationship very little (black-dashed line). Utilizing a more realistic model of the overlap between the knife-edge and the laser spot, as described in the text, reproduces the one-to-one relationship to within 10% of that assumed by Eq. (9). The shaded region represents ±10% of Eq. (9).

We note that although the ion gyroradius is large, of the order of a few mm, the MHD approximation is valid at all length scales considered, due to the small ion-ion collisional mean free path, of the order of 100 nm. Hence, we are in a regime where collisions dominate and the ions remain unmagnetized.

If the magnetic field is written as a sum of a uniform externally applied mean field and a fluctuating component, $\mathbf{B} = \mathbf{B_0} + \delta\mathbf{B}$, equation (10) becomes

$$\frac{\partial \delta\mathbf{B}}{\partial t} + \mathbf{u}.\nabla\delta\mathbf{B} = (\mathbf{B_0} + \delta\mathbf{B}).\nabla\mathbf{u} - (\mathbf{B_0} + \delta\mathbf{B})\nabla.\mathbf{u} + \eta\nabla^2\delta\mathbf{B}. \tag{11}$$

In the limit of small $R_m$, $\delta\mathbf{B} \ll \mathbf{B_0}$ and the diffusion term dominates over the nonlinear terms and the time derivative (quasistatic approximation). Then

$$\eta\nabla^2\delta\mathbf{B} = -\mathbf{B_0}.\nabla\mathbf{u} + \mathbf{B_0}\nabla.\mathbf{u}. \tag{12}$$

By taking the spatial Fourier transform we find

$$\eta k^2\delta\tilde{\mathbf{B}} = i(\mathbf{k}.\mathbf{B_0})\tilde{\mathbf{u}} - i\mathbf{B_0}(\mathbf{k}.\tilde{\mathbf{u}}), \tag{13}$$

where tildes denote Fourier-transformed quantities. Compared to the incompressible case[24,25], there is an additional term on the right-hand side. Let $\mathbf{B_0} = B_0\hat{\mathbf{x}}$, where $\hat{\mathbf{x}} = (1, 0, 0)$, and $\tilde{\mathbf{b}} = \eta k^2\delta\tilde{\mathbf{B}}/B_0$. Then equation (13) becomes

$$\tilde{\mathbf{b}} = ik_x\tilde{\mathbf{u}} - i\hat{\mathbf{x}}\mathbf{k}.\tilde{\mathbf{u}}. \tag{14}$$

If the turbulence is unaffected by $\mathbf{B_0}$ and is isotropic, then the velocity correlation tensor can be written as the sum of symmetric and antisymmetric parts, with the symmetric tensor itself written

as the sum of incompressible and compressible components:

$$\langle \tilde{u}_i \tilde{u}_j^* \rangle = I(k) \left( \delta_{ij} - \frac{k_i k_j}{k^2} \right) + C(k) \frac{k_i k_j}{k^2} + H(k) \epsilon_{ijl} \frac{k_l}{k}, \tag{15}$$

where $I(k)$, $C(k)$ and $H(k)$ are functions of $k$ only. From equation (14), we then find the magnetic-field correlation tensor:

$$\langle \tilde{b}_i \tilde{b}_j^* \rangle = k_x^2 \langle \tilde{u}_i \tilde{u}_j^* \rangle + \hat{\mathbf{x}}_i \hat{\mathbf{x}}_j k_m k_n \langle \tilde{u}_m \tilde{u}_n^* \rangle - k_x k_m \hat{\mathbf{x}}_i \langle \tilde{u}_m \tilde{u}_j^* \rangle - k_x k_n \hat{\mathbf{x}}_j \langle \tilde{u}_i \tilde{u}_n^* \rangle. \tag{16}$$

With the aid of equation (15), this becomes

$$\langle \tilde{b}_i \tilde{b}_j^* \rangle = I(k) k_x^2 \left( \delta_{ij} - \frac{k_i k_j}{k^2} \right) + H(k) k_x^2 \epsilon_{ijl} \frac{k_l}{k} + C(k) \left( \frac{k_x^2}{k^2} k_i k_j + k^2 \hat{\mathbf{x}}_i \hat{\mathbf{x}}_j - k_x \hat{\mathbf{x}}_i k_j - k_x \hat{\mathbf{x}}_j k_i \right). \tag{17}$$

In particular, the spectrum of any component of the field that is perpendicular to the mean field is

$$\langle |\tilde{b}_y|^2 \rangle = I(k) k_x^2 \left( 1 - \frac{k_y^2}{k^2} \right) + C(k) \frac{k_x^2 k_y^2}{k^2}, \tag{18}$$

and the spectrum of the component parallel to the magnetic field is

$$\langle |\tilde{b}_x|^2 \rangle = I(k) k_x^2 \left( 1 - \frac{k_x^2}{k^2} \right) + C(k) k^2 \left( 1 - \frac{k_x^2}{k^2} \right)^2 . \tag{19}$$

Averaging these spectra over angles, we find

$$\langle |\tilde{b}_y|^2 \rangle = \frac{k^2}{15} \left[ 4I(k) + C(k) \right], \tag{20}$$

$$\langle |\tilde{b}_x|^2 \rangle = \frac{k^2}{15} \left[ 2I(k) + 8C(k) \right]. \tag{21}$$

Solving these equations simultaneously, we find

$$I(k) = \frac{1}{2k^2} (8 \langle |\tilde{b}_y|^2 \rangle - \langle |\tilde{b}_x|^2 \rangle), \tag{22}$$

$$C(k) = \frac{1}{k^2} (2 \langle |\tilde{b}_x|^2 \rangle - \langle |\tilde{b}_y|^2 \rangle). \tag{23}$$

Thus, if we can measure or infer the angle-averaged spectra of two components of the perturbed inductive magnetic field, we can determine both the compressible and incompressible parts of the velocity spectrum.

Taking the trace of equation (15) and angle-averaging, we find that the total 1-D velocity power spectrum is given by

$$E(k) = 4\pi k^2 \langle |\tilde{\mathbf{u}}|^2 \rangle = 4\pi \left[ 2I(k) + C(k) \right] = 4\pi \left[ 7 \langle |\tilde{b}_y|^2 \rangle + \langle |\tilde{b}_x|^2 \rangle \right], \tag{24}$$

where the last expression follows from equations (22) and (23). Finally, the spectrum of $\tilde{\mathbf{b}}$ is related to the spectrum of $\delta\tilde{\mathbf{B}}$ by a factor of $k^4$, giving

$$E(k) \propto k^4 (7 \langle |\delta \tilde{B}_y|^2 \rangle + \langle |\delta \tilde{B}_x|^2 \rangle). \tag{25}$$

In the next section, we shall argue that wave-number spectra of magnetic fluctuations can be inferred from their frequency spectra. In our measurements, we have found that, within experimental error, the slopes of these frequency spectra were similar in the perpendicular and parallel directions: see Figure 4. Therefore, we may expect, for scaling purposes,

$$E(k) \propto k^2 M(k), \tag{26}$$

where $M(k) \propto k^2 \langle |\delta \tilde{B}_x|^2 \rangle \propto k^2 \langle |\delta \tilde{B}_y|^2 \rangle$ is the 1-D spectrum of the magnetic energy.

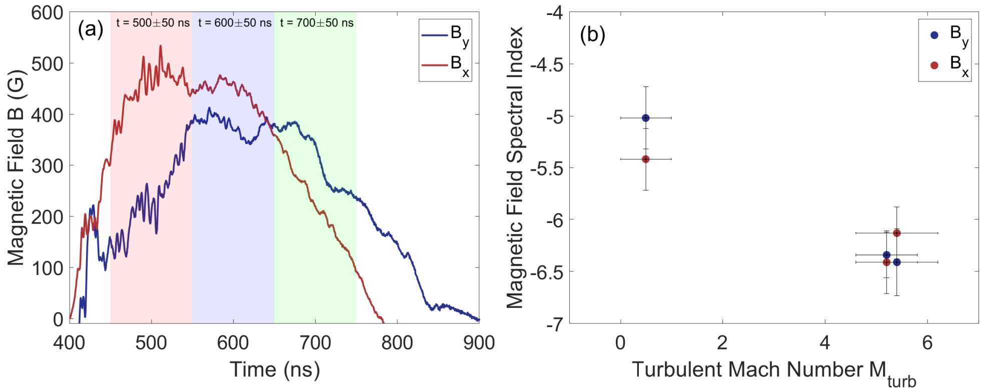


Supplementary Figure 4: **Temporal evolution of the magnetic field.** (a) Induction-loop measurements of the evolution of the $y$ and $x$ components of the magnetic field, i.e., of the fluctuations perpendicular and parallel to the direction of the external magnetic field. The shaded regions represent the extent over which the windowing function was applied in order to calculate the magnetic-field power spectra in the main paper. (b) Corresponding spectral index of the temporal magnetic field power spectrum, $M(\omega)$, obtained from the perpendicular and parallel magnetic field measurements. The error in the Mach number arise due to uncertainties in the measurement of the thermodynamic conditions of the plasma, as shown in Figure 2 of the main paper. The spectral index error bars represent the variation found by shifting the Fourier transform window by 20 ns in either direction.

## Relationship between frequency and wave-number spectra

The magnetic-field spectra discussed above are wave-number-resolved power spectra, whereas the induction loop of the B-dot probe measures frequency-resolved spectra. Had there been a mean flow past the B-dot probe, one would have expected the frequency spectrum measured by the probe's induction loop to follow the same scaling as the wave-number spectrum. This approximation does not hold in our case. However, a relation between the frequency and wavenumber spectra measured by the probe can still be found. Let us assume that there is a random flow of the largest eddies. This is justified in the experiment: from optical spectroscopy, we observe no shifts in the emission lines, which would otherwise indicate some mean flow at the outer scale. This random motion at the largest scale will then impose the stochastic version of the Taylor hypothesis, but there will be some effective, finite frequency interval between the outer scale and the scale where Taylor hypothesis kicks in. In that interval, a fixed probe would measure the "true" (Lagrangian) frequency spectrum.

In order to support the above considerations, we have performed large-scale simulations using the FLASH code[5–9]. To simplify the analysis, we have simulated a subsonic turbulent plasma,

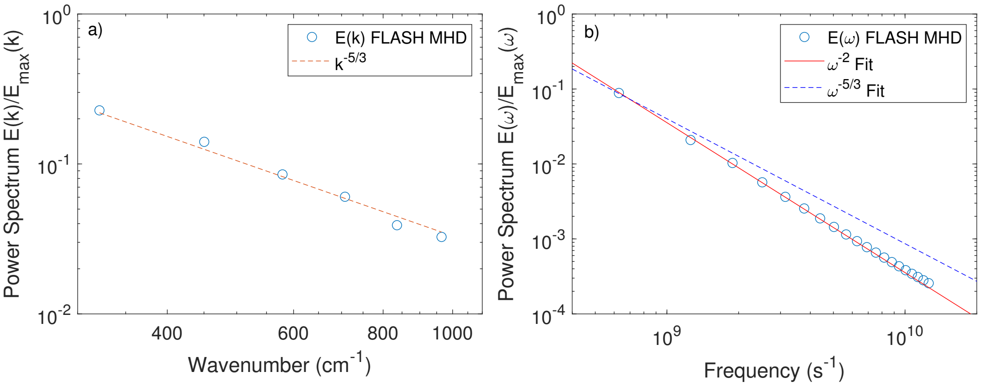


Supplementary Figure 5: **Numerical Validation of the Lagrangian Spectrum Approximation** (a) Simulations performed using the FLASH MHD code demonstrate that for a subsonic turbulent plasma, with no mean flow, the wave-number-resolved velocity power spectrum is consistent with a Kolmogorov power law. (b) For the same simulation, the frequency-resolved velocity power spectrum, obtained from the temporal evolution of the velocity at the center of the simulation, is shown to have an $\omega^{-2}$ slope, consistent with the Lagrangian frequency spectrum.

as described in Ref.[3], and focused our analysis at the flow collision region. In this case, we get a wave-number-resolved velocity power spectrum that is consistent with a Kolmogorov slope, as shown in Figure 5a. From the temporal profile of the velocity taken at a fixed point in the center of the interaction region, where the probe is located, we construct the frequency-resolved power spectrum. Figure 5b shows that this power spectrum is best fit by an $\omega^{-2}$ slope, supporting the expectation above that the probe measures a different power spectrum in frequency. For reference, we have also plotted a $\omega^{-\frac{5}{3}}$ slope, as would be expected in the presence of a large mean flow.

We note that the argument above may not hold at the smallest scales where a mean flow is effectively provided by the larger eddies in the plasma. Indeed, the spectrum shown in Figure 5b begins to deviate from an $\omega^{-2}$ slope at frequencies approximately 10 times the outer-scale frequency. In the experiment, this corresponds to frequencies beyond the resolution of the probe.

Let us now establish a relationship between this frequency spectrum and the wave-number spectrum of the underlying velocity field. We begin by assuming that velocity and magnetic-field increments at scale $\ell$ behave as power laws:

$$u(\ell) \sim \ell^{\alpha}, \quad B(\ell) \sim \ell^{\beta}. \tag{27}$$

The corresponding wave-number-resolved power spectra are

$$E(k) \sim k^{-(2\alpha+1)}, \quad M(k) \sim k^{-(2\beta+1)}. \tag{28}$$

From equation (26), we infer, for the two scaling exponents, the relationship

$$\beta = \alpha + 1. \tag{29}$$

The Lagrangian frequency corresponding to scale $\ell$ is $\omega \sim u(\ell)/\ell \sim \ell^{\alpha-1}$. Therefore, the velocity and magnetic-field increments associated with this frequency are

$$u(\omega) \sim \omega^{\frac{\alpha}{\alpha-1}}, \quad B(\omega) \sim \omega^{\frac{\beta}{\alpha-1}} \tag{30}$$

and the corresponding frequency spectra are

$$E(\omega) \sim \omega^{\frac{2\alpha}{\alpha-1}-1} \equiv \omega^{\kappa}, \quad M(\omega) \sim \omega^{\frac{2\beta}{\alpha-1}-1} \equiv \omega^{\xi}. \tag{31}$$

If we now denote the exponent of the velocity's wave-number spectrum $\sigma = -(2\alpha+1)$, we may use equations (29) and (31) to relate it to the exponents $\kappa$ and $\xi$ of the velocity and magnetic field's frequency spectra:

$$\sigma = -\frac{3\kappa+1}{\kappa-1}, \quad \sigma = -\frac{3\xi+5}{\xi-1}. \tag{32}$$

We see that the Kolmogorov power spectrum $\sigma = -5/3$ corresponds to $\kappa = -2$, in agreement with the FLASH simulations presented in Figure 5. For reference, blue the Kolmogorov power spectrum $\sigma = -5/3$ corresponds to $\xi = -5$, and the Burgers spectrum $\sigma = -2$ to $\xi = -7$.

**Extraction of temperature, density and turbulent broadening from optical spectroscopy**

The plasma temperature, mass density and turbulent velocity are inferred from the spatially resolved measurement of the Carbon II and Fluorine II spectral lines (see Figure 2 of the main manuscript). The spectroscopy was performed with an imaging spectrometer, coupled to an intensified CCD camera with a 20 ns gate width. The spatial resolution was set by the 50 $\mu$m slit of the spectrometer and the overall magnification was 0.17. Calibration was performed with a mercury lamp using the 404.6 nm, 407.8 nm and 435.8 nm lines giving 17.6 pixels/nm dispersion and $\lambda/\Delta\lambda \approx 2,000$ spectral resolution, where $\lambda$ is the measured wavelength.

For the fit, we utilized the three emission lines (located at approximately 425 nm, 427 nm, and 430 nm) that were consistently present across the extent of the plasma and above the noise level. Both the ratio and the width of Carbon and Fluorine emission lines were fitted using the collisional-radiative code PrismSPECT[10] assuming nonlocal thermodynamic equilibrium transition rates. The conditions achieved in this experiment are in the regime where the line ratio of the emission spectra is independent of electron density, because, for optically thin plasmas, the collisional deexcitation of atomic levels dominates over spontaneous radiative decay[21]. This occurs when the electron density $n_e \gg 1.7\times10^{14} T_e^{1/2}(\Delta E)^3$ cm$^{-3}$, where both $T_e$ and $\Delta E$ are given in electronvolts. For our plasma temperature ($T_e \approx 3$ eV) and optical transitions ($\Delta E \approx 2.9$ eV), we find this condition to be $n_e \gg 7\times10^{15}$ cm$^{-3}$, which is well satisfied in the regime of the experiment.

We performed 323 different simulations with mass densities ranging from 0.05 to $1\times10^{-5}$ g/cm$^3$ and temperatures spanning 2.8 to 4 eV. The simulations contain both natural and thermal broadening processes, which are strongly dependent on the ion species. In addition, a species-independent broadening was added to the spectra: we use this additional broadening to model the combined effect of the instrument resolution and turbulent motions within the plasma. Since the turbulent broadening affects all emission lines equally, we are able to deduce the temperature, density and turbulent velocity from the spectra. Since the calculated turbulent velocity is determined along the path of the emission line, the 3-D turbulent velocity reported in the main paper ($\mathrm{V_{turb}}$) is related to the measured 1-D turbulent velocity ($\mathrm{V_{1d}}$) by $\mathrm{V_{turb}} = \sqrt{3}\mathrm{V_{1d}}$, assuming isotropy[11]. The measured turbulent velocity is a spatially averaged quantity, where the size of the spatial average is given by the slit width of the spectrometer. Taking this into account along with the magnification of the system, we estimate the spatial averaging of this quantity to be over a $\sim 300$ $\mu$m region.

By performing a least-mean-squares fit between the experimental spectrum and the spectrum produced by PrismSPECT, we can produce a spatially resolved measurement of the electron temperature, mass-density and turbulent velocity at different times throughout the experiment. Example spectra for three distinct spatial regions, all taken at 600 ns, are shown in figure 6. We

note that the fluorine line at 430 nm corresponds to $3p - 3s$ transitions, while the one at 425 nm to $3d - 4f$ transitions. At temperatures $\gtrsim$3 eV, we would expect a larger fraction of fluorine ions to be in high-lying states, which explains why the line at 425 nm is more intense that the one at 430 nm. However, near the edge of the interaction region, the plasma cools down and gradients in the temperature are likely to affect the 430 nm line more. This would explain the poor fits seen in figure 6, where, away from the central region, the strong gradients cannot be fit by a spectrum corresponding to a single thermodynamic condition.

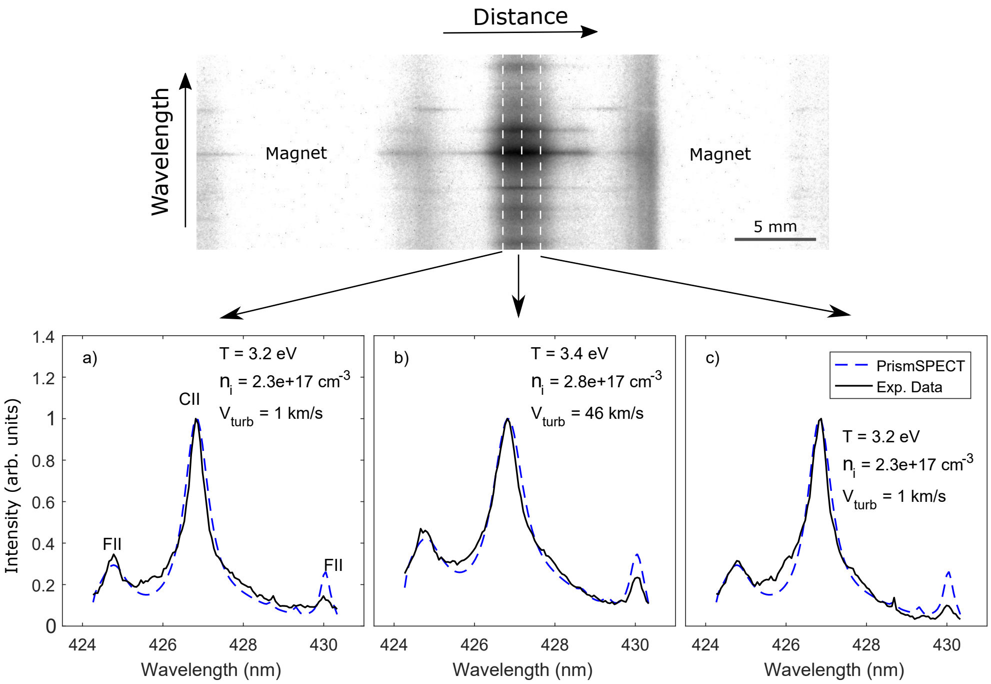


Supplementary Figure 6: **Spatial variation of the optical emission.** Example line-outs of the emission spectra across the spatial extent of the plasma at 600 ns. In the three bottom panels, we show the corresponding best-fit spectra from the collisional-radiative code PrismSPECT, along with the respective best-fit temperature, density and turbulent velocity.

## Calculation of electron density and ionization from optical interferometry

A Mach-Zehnder optical interferometer was used to measure the free-electron density in the colliding jets. The interferometer utilized the same probe beam as the Schlieren imaging system, a Photonic Solutions Powerlite Nd:YAG Laser. The laser wavelength was 532 nm with a ~5 ns pulse duration. The images were recorded on an intensified Princeton Instruments PI-MAX CCD camera with a 4 ns gate width. The analysis was carried out using the branch-cut technique[12] within the interferometry analysis software IDEA 1.7[13]. The high linear densities and large spatial variation present in the data precludes Abel inversion. Therefore, we obtain an estimate of the average electron density by assuming cylindrical symmetry and assuming the extent of the plasma in the $z$ direction to be the same as its extent in the $y$ direction.

Supplementary figure 7 gives an average electron density $n_e \sim 2 \times 10^{17}$ cm$^{-3}$ at $t = 400$ ns, increasing to a peak value $n_e \sim 6 \times 10^{17}$ cm$^{-3}$ by $t = 600$ ns. Combining this with the ion-density measurement obtained from the optical spectroscopy, we derive an average ionization state

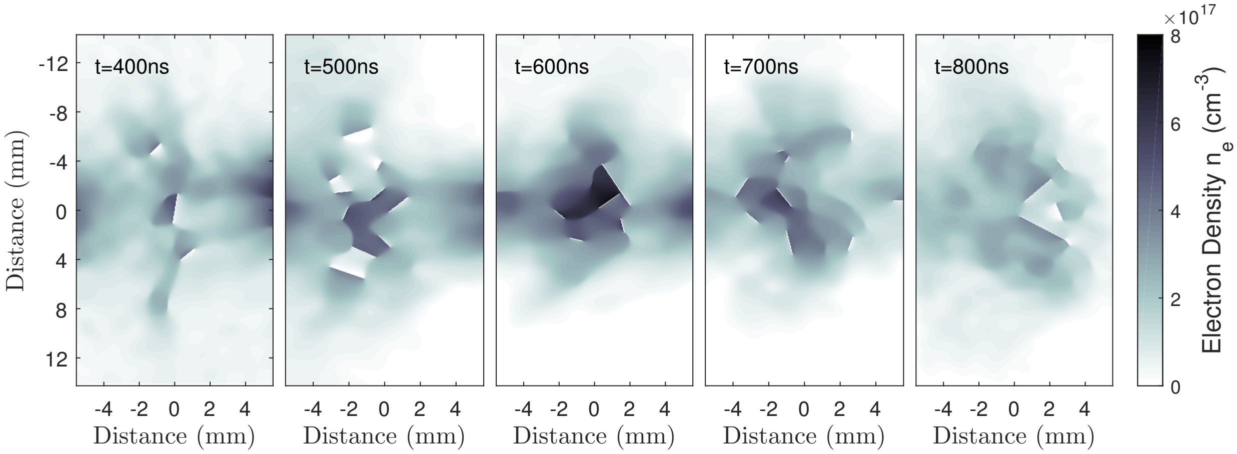


Supplementary Figure 7: **Temporal evolution of the electron density.** Images showing the temporal evolution of the electron density in the collided plasma, obtained via optical interferometry. The sharp lines present in the image indicate phase breaks in the interferometry due to high density gradients.

$Z^* \approx 1 - 2$. This is in agreement with the mean charge state calculated using the collisional–radiative code PrismSPECT, which for the temperatures and densities achieved in the experiment gives $Z^* \approx 1.6$.

## Supplementary References


[1] Meeker, D. C. Finite Element Method Magnetics, Version 4.2 (12Jan2016 Build), http://www.femm.info

[2] Everson, E. T., Pribyl, P., Constantin, C. G., Zylstra, A., Schaeffer, D., Kugland, N. L. & Niemann, C. Design, construction, and calibration of a three-axis, high-frequency magnetic probe (B-dot probe) as a diagnostic for exploding plasmas. *Review of Scientific Instruments* **80**, 113505 (2009).

[3] Tzeferacos, P., Rigby, A., Bott, A. F. A., Bell, A. R., Bingham, R., Casner, A., Cattaneo, F., Churazov, E. M., Emig, J., Fiuza, F., Forest, C. B., Foster, J., Graziani, C., Katz, J., Koenig, M., Li, C. -K., Meinecke, J., Petrasso, R., Park, H-S., Remington, B. A., Ross, J. S., Ryu, D., Ryutov, D., White, T. G., Reville, B., Miniati, F., Schekochihin, A. A., Lamb, D. Q., Froula, D. H. & Gregori, G. Laboratory evidence of dynamo amplification of magnetic fields in a turbulent plasma. *Nature Comms* **9** 591 (2018).

[4] Hutchinson, I. *Principles of Plasma Diagnostics* (Cambridge University Press, 2005).

[5] Fryxell, B., Olson, K., Ricker, P., Timmes, F. X., Zingale, M., Lamb, D. Q., MacNeice, P., Rosner, R., Truran, J. W. and Tufo, H. FLASH: An adaptive mesh hydrodynamics code for modeling astrophysical thermonuclear flashes. *Astrophysical Journal, Supplement* **131** 273 (2000).

[6] Dubey, A., Antypas, K., Ganapathy, M. K., Reid, L. B., Riley, K., Sheeler, D., Siegel, A. and Weide, K. Extensible component-based architecture for FLASH, a massively parallel, multiphysics simulation code. *Parallel Computing* **35(10-11)** 512 (2009)

[7] Tzeferacos, P., Fatenejad, M., Flocke, N., Graziani, C., Gregori, G., Lamb, D.Q., Lee, D., Meinecke, J., Scopatz, A. and Weide, K. FLASH MHD simulations of experiments that study shock-generated magnetic fields. *High Energy Density Physics* **17** 24-31 (2015).

[8] Graziani, C., Tzeferacos, P., Lee, D., Lamb, D. Q., Weide, K., Fatenejad, M. and Miller, J. The biermann catastrophe in numerical magnetohydrodynamics. *The Astrophysical Journal* **802(1)** 43 (2015).

[9] Tzeferacos, P., Rigby, A., Bott, A. F. A., Bell, A. R., Bingham, R., Casner, A., Cattaneo, F., Churazov, E. M., Emig, J., Flocke, N. Fiuza, F., Forest, C. B., Foster, J., Graziani, C., Katz, J., Koenig, M., Li, C. -K., Meinecke, J., Petrasso, R., Park, H-S., Remington, B. A., Ross, J. S., Ryu, D., Ryutov, D., Weide, K., White, T. G., Reville, B., Miniati, F., Schekochihin, A. A., Lamb, D. Q., Froula, D. H., Gregori, G. and Lamb, D. Q. Numerical modeling of laser-driven experiments aiming to demonstrate magnetic field amplification via turbulent dynamo. *Physics of Plasmas* **24(4)** 041404 (2017).

[10] MacFarlane, J., Golovkin, I., Woodruff, P., Kulkarni, S. & Hall, I. Simulation of the ionization dynamics of aluminum irradiated by intense short-pulse lasers. *Proc. Inertial Fusion and Sciences Applications 2003. Amer. Nucl. Soc.* 457-469 (2004).

[11] Inogamov, N. A. & Sunyaev, R. Turbulence in clusters of galaxies and X-ray line profiles. *Astronomy Letters* **29**, 791-824 (2003).

[12] Buckland, J. R., Huntley, J. M. & Turner, S. R. E. Unwrapping noisy phase maps by use of a minimum-cost-matching algorithm. *Applied Optics* **34**, 5100-5108 (1995).

[13] Hipp, M., Woisetschläger, J., Reiterer, P. & Neger, T. Digital evaluation of interferograms *Measurement* **36**, 53-66 (2004).

[14] Braginskii, S. Transport processes in a plasma. *Rev. Plasma Phys.* **1** 205 (1965).
